# Influence of Fish Consumption and ω-3 Supplementation on the ω-3 Index of Young Adults: A 2 × 2 Factorial Randomized Controlled Trial (YouFish Study)

**DOI:** 10.1016/j.tjnut.2025.10.010

**Published:** 2025-10-10

**Authors:** James E McMullan, Rohith Ajaya Kumar, Alison J Yeates, Philip J Allsopp, Maria S Mulhern, Edwin van Wijngaarden, J J Strain, Emeir M McSorley

**Affiliations:** 1 Nutrition Innovation Centre for Food and Health (NICHE), School of Biomedical Sciences, Ulster University, Coleraine, Northern Ireland, United Kingdom; 2 School of Medicine and Dentistry, University of Rochester, Rochester, NY, United States

**Keywords:** omega 3 PUFAs, fish consumption, omega 3 supplementation, young adults, cardiovascular disease

## Abstract

**Background:**

The omega-3 (n–3) index (O3I), reflecting the percentage of eicosapentaenoic acid (EPA) and docosahexaenoic acid (DHA) in erythrocyte membranes, is associated with reduced risk of cardiovascular disease (CVD). United Kingdom dietary guidelines recommend 2 portions of fish/week (280 g/wk) or supplementation of ∼500 mg EPA + DHA/d for non/low-fish consumers; however, the impact of these recommendations on the O3I is unclear.

**Objectives:**

The aim of this study was to explore the influence of the current guidance for fish consumption and ω-3 supplementation on the O3I among young adults.

**Methods:**

Healthy adults aged 18–30 y (*n =* 40) with low-fish intake and O3I <6% were randomly assigned to receive either 2 fish (1 portion oily fish and 1 portion white fish) or 2 nonfish meals per week as well as a daily 700 mg EPA+DHA supplement capsule or placebo capsule for 8-wk in a 2×2 factorial design. The effects on lipid profiles and high-sensitivity C-reactive protein (hs-CRP) were also examined.

**Results:**

Consumption of 2 portions of fish/week and ω-3 supplementation resulted in a significant mean % increase in O3I of 2.27% ± 0.82% and 2.03% ± 0.88%, respectively. Both interventions also significantly increased total erythrocyte n–3 polyunsaturated fatty acids (n–3 PUFAs) (+3.11% ± 1.79% and +2.00% ± 1.24%) and lowered total n–6 PUFAs (–1.94% ± 1.65% and –2.60% ± 1.16%) (all false discovery rate *P <* 0.05). There were no significant effects on blood lipids or hs-CRP.

**Conclusions:**

In support of current dietary guidelines for fish consumption and ω-3 intake, 2 portions of fish/week or ω-3 supplementation are effective strategies for increasing the O3I. These findings support the efficacy of current public health recommendations for fish consumption and ω-3 intake as early dietary interventions to reduce CVD risk through increasing the O3I among young adults.

This trial was registered at www.clinicaltrials.gov as NCT06729229.

## Introduction

The ω-3 index (O3I) refers to the percentage of the n–3 (ω-3) PUFAs, EPA (C20:5 n–3), and DHA (C22:6 n–3) in erythrocyte cell membranes, with respect to total fatty acids. Consistent epidemiologic evidence now supports a substantive role for the O3I as a modifiable risk factor for cardiovascular disease (CVD) where higher levels are associated with a reduced risk of cardiac events [1]. An O3I >8% is proposed to be the optimal index and confer the greatest level of cardioprotection, whereas <4% is considered to be the high-risk category [2].

Globally, dietary recommendations for n–3 PUFAs vary; however, it has been suggested that an intake of 250 mg EPA + DHA per day is sufficient for the primary prevention of CVD [3]. Fish is the richest source of the n–3 PUFAs within the diet. Current United Kingdom dietary guidelines advise the consumption of 2 portions of fish per week (equivalent to 280 g/wk), with ≥1 portion being oily (140 g), which would contribute ∼450 mg EPA + DHA per day [4]. Concerningly, recent evidence has shown that current fish intakes are well below these recommendations [5]. The reasons for the avoidance of fish at a population level remain unclear but may be owing to a general ambiguity surrounding the potential risks of fish consumption from contaminants [6,7]. Many who have adopted a cautionary approach toward fish consumption have been shown to perceive ω-3 supplementation as a safe method of meeting nutritional requirements [8]. Professional bodies have noted that a daily ω-3 supplement, equating to ∼500 mg EPA + DHA per day, is beneficial in increasing n–3 PUFA intakes among those who may exclude dietary sources, such as fish [9,10].

Previous observational evidence has noted both regular consumption of fish and ω-3 supplementation to be 2 of the strongest predictors of the O3I [11,12]; nevertheless, findings from intervention studies vary in terms of the magnitude of effect, owing to differences in dosage and duration. One small study among healthy adults reported an absolute increase of 2.5% in the O3I with supplementation of 556 mg EPA + 237 mg DHA per day for 5 mo [13], whereas another study reported a smaller absolute increase of 1.6% with supplementation of 140 mg EPA + 560 mg DHA per day for 8 wk [14]. Currently, fewer studies have intervened with fish to determine the degree to which intakes improve the O3I. One study reported a 1.01% absolute increase in the O3I with 150 g of oily fish twice per week [8] whereas another noted a smaller absolute increase of 0.6% with 80–100 g oily fish 3 times per week [15].

Concerningly, the O3I across the United Kingdom population remains low, with United Kingdom adults estimated to have an O3I of ∼5.60% [12]. Notably, fish consumption appears to be the lowest among young adults with only 15.6% aged 20–29 y meeting the current oily fish recommendations, which may have implications for CVD development [6]. Early dietary interventions, such as increasing n–3 PUFA intakes to increase the O3I during early adulthood, may prove to be an effective intervention in the early prevention of future CVD events [12].

Although several studies have shown both fish intakes and ω-3 supplementation to increase the O3I, few have matched the current United Kingdom recommendations for fish consumption or the current guidance surrounding supplementation. Therefore, the aim of the current study was to explore the influence of the current guidance for fish consumption and ω-3 supplementation on the O3I among young adults with low-habitual fish intake. Secondary analysis examined the effect of fish and ω-3 supplementation on other markers of CVD including blood lipid concentrations and high-sensitivity C-reactive protein (hs-CRP).

## Methods

### Study design and population

The YouFish Study [registered at www.clinicaltrials.gov (NCT06729229)] was an 8-week 2×2 factorial randomized controlled trial with the overall aim of investigating the effect of fish consumption and ω-3 supplementation on the O3I among young adults aged between 18 and 30 y. Male and female participants were recruited from Ulster University, Coleraine, United Kingdom and the surrounding area from November 2024 until January 2025. Inclusion criteria included: healthy males or females aged between 18 and 30 y, low consumers of fish (<2 portions of fish per month), willing to consume 2 or no portions of fish per week, nonconsumers of fish oil supplements, not allergic to seafood, and an O3I <6%. All participants provided written informed consent before commencing the study. The study was approved by Ulster University Research Ethics Committee (REC/24/0005) and conducted in accordance with the 1964 Declaration of Helsinki and its amendments.

A 2×2 factorial design was employed in this study giving the ability to test the effect of 2 independent treatments (fish and ω-3 supplements) on the O3I assuming independent intervention effects. Participants were randomly allocated to one of the following 4 intervention groups: *1)* 2 fish meals and ω-3 supplement, *2)* 2 fish meals and placebo supplement, *3)* 2 control meals (no fish) and ω-3 supplement, or *4)* 2 control meals (no fish) and placebo supplement. Participants were allocated to the supplement groups in a double-blind manner. Participants were randomly assigned to one of the 4 intervention groups by an independent Clinical Trial Manager using a web-based procedure consisting of block sizes of 5×8 and an equal allocation sequence of 2:2:2:2 across the 4 groups within each block.

### Screening

All those interested and who met the initial eligibility criteria were invited to a screening appointment where they completed a screening questionnaire and provided a dried blood spot sample via a single finger prick. Dried blood spot samples were then sent to OmegaQuant Europe, University of Sterling, Scotland, United Kingdom (https://omegaquant.com/) for the measurement of the O3I to ensure participants were low consumers of fish and not consuming any supplements containing n–3 PUFAs. Only participants with an O3I <6% were deemed eligible for inclusion within the study. Power calculations were based on a 2×2 factorial design using a fixed-effects 2-way analysis of variance to test the main effects of fish consumption and ω-3 supplementation. Power calculations were based on observing a 2.0% increase in the O3I between levels of each factor and a within-cell SD of 1.09 [13]. Calculations were performed using Power Analysis Sample Size software at a 2-sided significance level of 0.05. A total of 5 participants per treatment group would achieve ∼91% power for each main effect. To ensure 5 participants completed each of the 4 treatment groups, a total of 10 participants were required per group.

### Intervention period

Eligible participants were invited to attend 2 sampling appointments (baseline and postintervention) at the Human Intervention Studies Unit at Ulster University beginning in January 2025. At each time point, participants provided biological samples including blood, anthropometric measurements, and information on habitual diet and lifestyle. Study participants were then provided with lunch twice per week at the Human Intervention Studies Unit, where they received either 2 portions of fish or 2 portions of chicken. Those allocated to the fish intervention were provided with 2 lunch dishes per week containing a total of 280 g fish per week (140 g oily fish and 140 g white fish) [4] for 8 wk. Fish lunches included a salmon salad and breaded whiting fillet. Those allocated to the nonfish meal were provided with 2 lunch meals each containing 140 g of chicken. Nonfish lunches included chicken salad and breaded chicken fillet.

Participants were also randomly assigned to an ω-3 capsule or a placebo capsule daily for the duration of the 8-wk intervention. The active ω-3 supplement contained 400 mg EPA and 300 mg DHA in triglyceride form. Dosage was based on the guidance surrounding ω-3 supplementation, which suggests a daily ω-3 supplement, equating to ∼500 mg EPA + DHA per day, is sufficient for those who do not include fish within the diet [9,10]. Those in the placebo supplement group were provided with a visually identical placebo capsule containing corn oil. The active ω-3 supplement and the placebo capsule were provided by EPAX Norway (https://www.epax.com/), and both sets of capsules were in identical packaging to maintain study blinding.

The fish-based dietary intervention and ω-3 supplement were not dose matched but were designed to reflect current United Kingdom guidance for fish consumption and ω-3 supplementation [4, [9], [10]]. Estimated weekly intakes of EPA, DHA, and total EPA+DHA from both interventions are provided in Supplemental Table 1. On the basis of published nutrient composition data [16], the weekly intake from 2 portions of fish was estimated at 0.826 g EPA and 1.568 g DHA, giving a total of 2.40 g EPA + DHA per week. The ω-3 supplement provided 2.80 g EPA and 2.10 g DHA per week, giving a total of 4.90 g EPA + DHA per week.

Compliance with the lunch-time meals was assessed by weighing each meal before serving (standard portion weights) and recording the weight of any leftovers after consumption. The amount consumed was calculated as the served weight minus the leftover weight, and compliance was expressed as the percentage of the given portion consumed across the intervention period. Compliance with the capsule intervention was assessed by counting the number of capsules returned at the end of the 8-wk intervention and comparing this with the number required to be taken (one capsule per day for 56 d), with adherence expressed as the percentage of capsules consumed.

### Blood sampling and anthropometric measurements

Fasting blood samples collected at baseline and postintervention were processed to obtain serum, plasma and red blood cells by centrifugation at 3000 ×*g* for 15 min at 4°C. All aliquots were frozen and stored at –80°C until batch analysis. Body weight (to the nearest 0.1 kg) was measured using TANITA digital scales (TANITA). Height (meters) was measured and recorded to the nearest centimeter using a calibrated stadiometer and BMI (kg/m^2^) was then calculated.

### O3I and fatty acid analysis

Red blood cell samples were shipped to OmegaQuant Europe, University of Sterling, Scotland, United Kingdom (https://omegaquant.com/) for the analysis of baseline and postintervention erythrocyte membrane fatty acid content and the O3I using capillary column gas chromatography [17]. Erythrocyte membrane fatty acid composition was expressed as a weight percentage of total identified fatty acids after response factor correction. The O3I was then calculated as the weight percentage of EPA + DHA with respect to total fatty acids.

### Biomarker analyses

Biomarkers known to be influenced by ω-3 PUFA were analyzed at both timepoints. Plasma lipids were analyzed using the RX Daytona + Analyzer. Plasma triglycerides were analyzed using Glycerol Phosphate Oxidase - Peroxidase (GPO-PAP) colorimetric end-point assay and HDL cholesterol and total cholesterol were analyzed using Cholesterol Oxidase-Peroxidase-Phenol-Aminoantipyrine (CHOD-PAP) colorimetric end-point assays. LDL cholesterol was calculated using the Friedewald formula [total cholesterol – HDL – (triglycerides/2.2)]. The RX Daytona + Analyzer was also used to measure hs-CRP in serum samples.

### Dietary intake and general health

At baseline, participants were asked to complete a 4-d food diary to assess habitual dietary intakes before the intervention. Food diaries were completed over 4 consecutive days (2 weekdays and 2 weekend days) and returned at the first lunch appointment. Portion sizes were estimated using household measurements, and participants were asked to record brand names where possible. Dietary intakes were then quantified using Nutritics software (Nutritics Ltd, 2018). Participants completed a health and lifestyle questionnaire to provide information on general health, including supplement usage, personal medical history, and smoking and alcohol consumption, as well as demographic and socioeconomic data. Participants were asked to refrain from consuming any other fish or ω-3 supplements for the duration of the intervention period but to otherwise follow their habitual diet and lifestyle. At each lunch-time meal, participants were asked if they had eaten any fish in addition to the provided study portion. If additional fish was consumed, details including the type and quantity eaten were obtained. No participants reported consuming any additional fish outside of the intervention; therefore, this was not accounted for within the analysis.

### Statistical analysis

**FIGURE 1:**
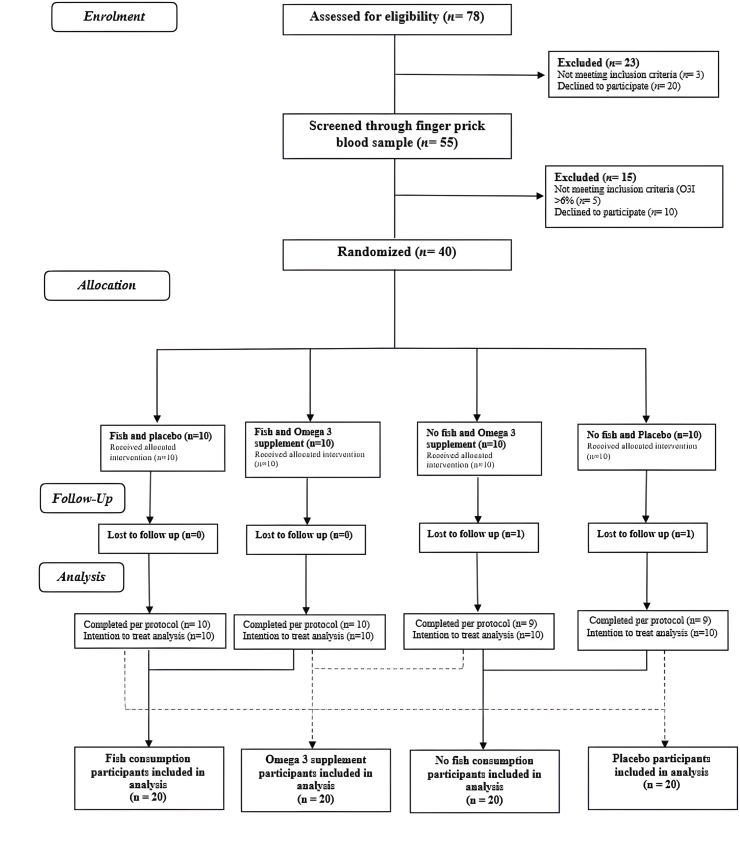
CONSORT flow diagram for study design. O3I, omega 3 index.

Statistical analysis was conducted using Statistical Package for Social Sciences (SPSS, version 29.0, IBM). All statistical analyses were completed using intention to treat (ITT), as specified a priori. For ITT analyses, given the small amount of missing data (<5%) and smaller sample size, baseline values of participants lost to follow up were entered as postintervention values [18,19], consistent with approaches used within other dietary interventions [20]. To minimize potential bias from single imputation, analyses were also completed per protocol. Results from ITT and per-protocol analyses did not differ and ITT findings are reported. Analysis compared *1)* all those randomly assigned with the fish intervention compared with all those not allocated with the fish intervention and *2)* all those randomly assigned with the ω-3 supplement compared with all those not allocated to the ω-3 supplement (Figure 1). Data were checked for normality using Shapiro–Wilk test and log transformed, where appropriate, to approximate normality before analysis. Descriptive statistics were used to summarize participant characteristics, and all data are expressed as mean ± SD for normally distributed data and median (IQR) for non-normally distributed data. Differences in baseline characteristics between the fish and supplement groups and their respective control groups were analyzed using independent samples *t* test or Mann–Whitney *U* test where appropriate and χ^2^ for categorical data.

Factorial analysis of covariance (ANCOVA) was used to examine the main effects of fish consumption and ω-3 supplementation on the O3I, adjusting for age, sex, BMI, baseline O3I, and the other intervention factor. Covariates were selected a priori based on factors known to influence the O3I. Alcohol consumption was considered as a potential covariate due to observed baseline differences between groups; however, it was not significantly associated with the primary or secondary outcomes. Only covariates selected a priori (age, sex, BMI, and relevant baseline concentrations) were retained in the final models. Differences in the proportions of participants within O3I categories were assessed using Fisher’s exact test because of the small numbers in each group. Factorial ANCOVA, adjusting for age, sex, BMI, the relevant baseline value, and the other intervention factor, was also used to assess the effects of both interventions on blood lipid profiles and hs-CRP. For each outcome, a 2-way ANCOVA model was first fitted including both main effects (dietary fish intervention and ω-3 supplementation) and their interaction term. As no significant interactions were observed for the primary or secondary outcomes, final models only included the main effects. A *P* value <0.05 was considered statistically significant. To account for multiple testing, *P* values were adjusted using the Benjamini–Hochberg false discovery rate (FDR) procedure, with a FDR set at 5%. Both raw and FDR-adjusted *P* values are reported.

Sensitivity analysis was performed using the 4-arm model that retained the original randomly assigned groups. ANCOVA with Least Significant Difference (LSD) for post hoc comparison was used adjusting for age, sex, BMI, and relevant baseline concentrations.

## Results

The CONSORT flow diagram outlines the number of participants screened and recruited onto the study, and those who completed the intervention (Figure 1). A total of 55 apparently healthy males and females were screened for eligibility within the study. Five participants were excluded owing to an O3I >6% and 10 withdrew before randomization. Subsequently, 40 young adults (29F, 11M) completed a baseline appointment and were randomly assigned to one of 4 intervention groups. Two participants withdrew from the study and were lost to follow up (Figure 1). Overall, compliance to lunches was high with 99% and 98% adherence for the fish and no fish groups, respectively. Compliance to supplementation was also high with 94.8% adherence within the supplement group and 90.7% within the placebo group. There was no statistically significant interaction effect on the primary outcome (O3I) between the fish-based dietary intervention and ω-3 supplementation over 8 wk [interaction coefficient: 0.81 (95% confidence interval: –0.25,1.87); *P =* 0.130]. There was also no significant interaction effect on any of the secondary outcomes (all *P >* 0.05). Therefore, analyses of primary and secondary outcomes are presented for the main effects of each intervention separately.

Baseline characteristics for the entire cohort and for intervention groups separately are shown in Table 1. The median (IQR) age for this cohort was 21.0 (20.0–24.5) y. Participants had a mean ± SD O3I of 5.06% ± 0.60%, with the majority of all participants (*n* = 38, 95.0%) classified within the medium O3I CVD risk category (4%–8%), whereas 5.0% (*n* = 2) were in the high-risk category (<4%). At baseline, a significantly greater proportion of those in the ω-3 supplement group reported to consume alcohol when compared with the placebo group. No other significant differences in baseline characteristics were observed between groups (Table 1). After FDR correction, there were also no significant differences in dietary PUFA intakes among treatment groups (all FDR *P >* 0.05) (Supplemental Table 2).

**TABLE 1:** Characteristics of YouFish study participants at baseline for the whole cohort, and according to intervention group.

	Whole group (*n =* 40)	Fish (*n =* 20)	No Fish (*n =* 20)	Supplement (*n =* 20)	Placebo (*n =* 20)
Age (y)	21.0 (20.0, 24.8)	21.0 (21.0, 24.5)	22.0 (20.0, 24.8)	21.5 (20.3, 25.0)	21.0 (20.0, 23.8)
Weight (kg)	64.8 (59.5, 77.8)	64.7 (58.8, 73.8)	67.3 (60.7, 84.3)	63.6 (58.3, 79.6)	67.9 (61.4, 77.5)
Height (m)	1.69 ± 0.08	1.69 ± 0.09	1.69 ± 0.07	1.68 ± 0.07	1.71 ± 0.08
BMI (kg/m^2^)	24.14 ± 3.55	23.40 ± 2.72	24.87 ± 4.16	24.32 ± 3.80	23.96 ± 3.31
Body fat (%)	15.5 ± 7.5	15.3 ± 7.3	15.7 ± 7.9	15.6 ± 6.6	15.4 ± 8.5
Gender					
Female	29 (72.5)	16 (80.0)	13 (65.0)	16 (80.0)	13 (65.0)
Male	11 (27.5)	4 (20.0)	7 (35.0)	4 (20.0)	7 (35.0)
Consumes alcohol					
Yes	30 (75.0)	13 (65.0)	17 (85.0)	18 (90.0)	12 (60.0)
No	10 (25.0)	7 (35.0)	3 (15.0)	2 (10.0)	8 (40.0)
Smoker					
Yes	1 (2.5)	0 (0.0)	1 (5.0)	0 (0.0)	1 (5.0)
No	39 (97.5)	20 (100.0)	19 (95.0)	20 (100.0)	19 (95.0)
O3I (%)	5.06 ± 0.60	5.00 ± 0.66	5.12 ± 0.56	5.06 ± 0.57	5.06 ± 0.65
ALA (%)	0.17 (0.13, 0.24)	0.18 (0.14, 0.28)	0.16 (0.13, 0.20)	0.17 (0.13, 0.22)	0.17 (0.13, 0.28)
EPA (%)	0.57 (0.47, 0.68)	0.56 (0.44, 0.68)	0.58 (0.49, 0.69)	0.59 (0.50, 0.70)	0.54 (.45, 0.68)
DPA (%)	2.83 (2.42, 3.06)	2.79 (1.85, 3.04)	2.89 (2.62, 3.22)	2.81 (2.52, 3.23)	2.86 (1.63, 2.97)
DHA (%)	4.25 (3.65, 4.75)	3.98 (3.26, 4.75)	4.43 (3.96, 4.87)	4.36 (3.93, 4.84)	4.00 (3.22, 4.70)
					
Total n-3	7.99 (7.11, 8.44)	7.43 (6.29, 8.39)	7.88 (7.47, 8.60)	8.16 (7.34, 8.60)	7.61 (6.08, 8.39)
O3I category					
High (<4%)	2 (5.0)	2 (10.0)	0 (0.00)	0 (0.00)	2 (10.0)
Medium (4%–8%)	38 (95.0)	18 (90.0)	20 (100.0)	20 (100.0)	18 (90.0)
Low (>8%)	0 (0.00)	0 (0.0)	0 (0.0)	0 (0.0)	0 (0.0)

Data presented as mean ± SD or median (IQR), where IQR is the 25th and 75th percentile; or *n* (%) where appropriate.

*P* value for significant difference between intervention groups at baseline as determined using independent samples t test for parametric data and Mann–Whitney U test for nonparametric data, or Chi square as appropriate [all *P* > 0.05, with the exception of alcohol consumption, with a significantly greater proportion of those in the ω-3 supplement group reported to consume alcohol when compared with the placebo group (*P* = 0.028)].

Abbreviations: ALA, alpha linoleic acid; DPA, docosapentaenoic acid; O3I, ω 3 index.

**FIGURE 2:**
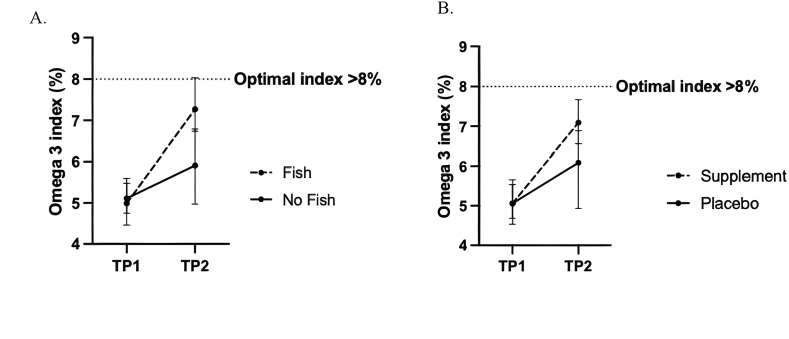
Mean ± SD change in O3I in (A) fish intervention and (B) ω-3 supplement over time relative to control groups. TP, timepoint.

**FIGURE 3:**
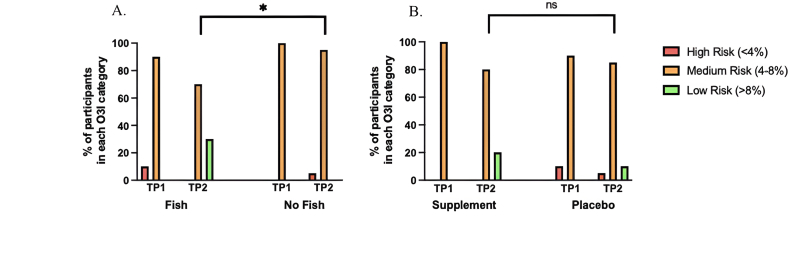
Effect of (A) fish and (B) ω-3 supplementation on the proportion of participants in each ω-3 index category. (A) ∗Significantly greater proportion of participants in the low-risk O3I category (>8%) in the fish group compared with those in the no fish group (30.0% compared with 0.0%, respectively, *P* = 0.021). (B) No significant association between ω-3 supplementation and postintervention ω-3 index categories (*P* = 0.428). Between-group postintervention differences assessed using Fisher’s exact test, sig *P <* 0.05. O3I, ω 3 index; TP, timepoint; ns, nonsignificant.

Figure 2 displays the mean change in the O3I over time within the fish and ω-3 supplement groups. Consumption of 2 portions of fish per week resulted in a mean ± SD increase in the O3I (from baseline) of 2.27% ± 0.82% (5.00% ± 0.66% compared with 7.27% ± 1.13%). Similarly, ω-3 supplementation resulted in a mean ± SD increase (from baseline) of 2.03% ± 0.88% (5.06 ± 0.57 compared with 7.09 ± 0.87). TABLE 2, TABLE 3 summarize the effect of the interventions on the O3I and erythrocyte fatty acid concentrations at week 8. For the first principal comparison, fish compared with no fish, consumption of 2 portions of fish per week significantly increased the O3I and erythrocyte concentrations of DHA and total n–3 PUFAs when compared with those consuming no fish (all FDR *P <* 0.05). There were no significant differences in EPA concentrations between the fish and control group (FDR *P* = 0.106). There were also no significant differences in the individual n–6 PUFAs, linoleic acid (LA; 18:2n–6) or arachidonic acid (AA; 20:4n–6) concentrations between groups; however, consumption of 2 portions of fish per week resulted in significantly lower total n–6 PUFAs when compared with the no fish group (FDR *P* = 0.042) (Table 2). Following dietary intervention with fish, the proportion of participants in the low-risk O3I category (>8%) was significantly greater than those in the no fish group (*n* = 6, 30.0% compared with *n* = 0, 0.0%, respectively, *P* = 0.021) (Figure 3).

**TABLE 2:** The effect of dietary intervention with fish on ω-3 index and red blood cell fatty acid concentrations.

	Fish (*n =* 20)		No fish (*n =* 20)		*P* value^1^	*q* value^1^	Partial Eta squared
	Baseline	Postintervention	Baseline	Postintervention			
O3I (%)	5.00 ± 0.66	7.27 ± 1.13	5.12 ± 0.56	5.90 ± 1.03	<0.001∗	<0.001∗	0.377
LA (%)	14.74 ± 4.50	12.01 ± 1.53	12.93 ± 2.59	12.36 ± 2.56	0.299	0.411	0.031
AA (%)	15.71 ± 2.61	16.56 ± 1.00	16.81 ± 1.14	16.84 ± 1.90	0.696	0.696	0.005
ALA (%)	0.30 ± 0.31	0.16 ± 0.04	0.18 ± 0.07	0.18 ± 0.04	0.072	0.113	0.097
EPA (%)	0.56 ± 0.14	1.16 ± 0.41	0.59 ± 0.14	0.97 ± 0.45	0.058	0.106	0.056
DPA (%)	2.49 ± 0.72	3.08 ± 0.26	2.78 ± 0.62	3.13 ± 0.64	0.426	0.521	0.019
DHA (%)	4.03 ± 0.86	6.10 ± 0.84	4.34 ± 0.71	4.85 ± 0.85	<0.001∗	<0.001∗	0.372
Total n–6	36.74 ± 1.20	34.80 ± 1.47	36.23 ± 1.25	35.65 ± 1.60	0.019∗	0.042∗	0.151
Total n–3	7.39 ± 1.99	10.50 ± 1.24	7.88 ± 1.14	9.12 ± 1.66	0.003∗	0.011∗	0.230
AA:LA ratio	1.18 ± 0.42	1.40 ± 0.21	4.72 ± 0.95	4.10 ± 1.16	0.649	0.696	0.241
EPA:ALA ratio	2.91 ± 1.68	7.91 ± 3.55	1.36 ± 0.32	1.42 ± 0.30	0.013∗	0.036∗	0.006

Data presented as mean ± SD.

Abbreviations: AA, arachidonic acid; ALA, alpha linoleic acid; DPA, docosapentaenoic acid; FDR, false discovery rate; LA, linoleic acid; O3I, ω-3 index.

1 Between-group postintervention differences assessed using analysis of covariance adjusting for age, sex, BMI, baseline concentrations, and ω 3 supplement intervention ∗Sig FDR corrected *q*< 0.05.

**TABLE 3:** The effect of ω-3 supplementation on ω-3 index and red blood cell fatty acid concentrations.

	Supplement (*n =* 20)		Placebo (*n =* 20)		*P* value^1^	*q* value	Partial Eta squared
	Baseline	Postintervention	Baseline	Postintervention			
O3I (%)	5.06 ± 0.57	7.09 ± 0.87	5.06 ± 0.65	6.09 ± 1.42	0.007∗	0.018∗	0.366
LA (%)	12.73 ± 2.86	11.54 ± 1.15	14.95 ± 4.22	12.83 ± 2.51	0.171	0.235	0.041
AA (%)	17.11 ± 1.47	16.77 ± 0.96	15.42 ± 2.83	16.64 ± 1.93	0.338	0.372	0.024
ALA (%)	0.20 ± 0.13	0.16 ± 0.03	0.28 ± 0.30	0.18 ± 0.05	0.300	0.367	0.021
EPA (%)	0.60 ± 0.15	1.34 ± 0.36	0.56 ± 0.13	0.79 ± 0.31	<0.001∗	<0.001∗	0.464
DPA (%)	2.80 ± 0.51	3.31 ± 0.34	2.47 ± 0.79	2.89 ± 0.52	0.158	0.235	0.058
DHA (%)	4.37 ± 0.61	5.75 ± 0.68	4.00 ± 0.92	5.20 ± 1.28	0.148	0.235	0.062
Total n–6	36.71 ± 1.28	34.70 ± 1.35	36.27 ± 1.99	35.76 ± 1.64	0.002∗	0.007∗	0.338
Total n–3	7.96 ± 0.79	10.56 ± 0.87	7.30 ± 1.43	9.07 ± 1.84	0.008∗	0.018∗	0.328
AA:LA ratio	4.67 ± 0.70	3.32 ± 0.39	5.18 ± 1.21	4.16 ± 1.19	0.603	0.603	0.011
EPA:ALA ratio	1.40 ± 0.28	1.47 ± 0.20	1.14 ± 0.42	1.35 ± 0.29	<0.001∗	<0.001∗	0.394

Data presented as mean ± SD.

Abbreviations: AA, arachidonic acid; ALA, alpha linoleic acid; DPA, docosapentaenoic acid; LA, linoleic acid; O3I, ω-3 index.

1 Between-group postintervention differences assessed using analysis of covariance adjusting for age, sex, BMI, baseline concentrations, and dietary fish intervention, ∗Sig *q* < 0.05.

For the second principal comparison, ω-3 supplementation compared with placebo, similar results were observed. Ω-3 supplementation resulted in significant increases in the O3I and erythrocyte concentrations of EPA and total n–3 PUFAs when compared with those in the placebo group (all FDR *P <* 0.05). There were no significant differences in DHA between the supplement and placebo group (FDR *P* = 0.235). There were also no significant differences in LA or AA concentrations, albeit similar to the fish intervention, supplementation resulted in significantly lower total n–6 PUFAs when compared with the placebo group (FDR *P*= 0.007) (Table 3). Postintervention, the proportion of participants in the low-risk O3I category (>8%) was greater among the ω-3 supplement group (*n* = 4, 20.0%) when compared with the placebo group (*n* = 2, 10.0%), although, this did not reach statistical significance (*P* = 0.428) (Figure 3).

TABLE 4, TABLE 5 display the effect of fish consumption and ω-3 supplementation on secondary outcomes including lipid profiles and hs-CRP. The majority of participants had mean cholesterol and CRP concentrations within the recommended clinical range at both timepoints with only *n* = 7 having total cholesterol >5.0 mmol/L and *n* = 3 having CRP values >3.0 mg/L. The fish intervention had no significant effect on lipid profiles or hs-CRP (Table 4). Neither was there any significant effect of ω-3 supplementation on lipid profiles or hs-CRP when compared with the placebo group (Table 5).

**TABLE 4:** The effect of dietary intervention with fish on blood lipid concentrations and hs-CRP.

	Fish (*n =* 20)		No fish (*n =* 20)		*P* value^1^	*q* value	Partial Eta squared
	Baseline	Postintervention	Baseline	Postintervention			
Total Chol (mmol/l)	4.17 ± 0.87	4.21 ± 0.92	4.53 ± 0.75	4.53 ± 0.81	0.721	0.799	0.002
HDL (mmol/L)	1.49 ± 0.32	1.41 ± 0.30	1.31 ± 0.25	1.26 ± 0.26	0.819	0.819	0.005
LDL (mmol/L)	2.29 ± 0.86	2.06 ± 0.75	2.88 ± 0.79	2.56 ± 0.79	0.644	0.799	0.007
Trig (mmol/L)	0.70 ± 0.25	0.66 ± 0.32	0.93 ± 0.42	0.96 ± 0.45	0.247	0.494	0.102
TC:HDL	2.89 ± 0.65	3.05 ± 0.61	3.52 ± 0.72	3.70 ± 0.80	0.601	0.799	0.026
Trig:HDL	0.49 ± 0.18	0.48 ± 0.18	0.76 ± 0.43	0.84 ± 0.53	0.133	0.494	0.103
Non-HDL (mmol/l)	2.69 ± 0.79	2.80 ± 0.78	3.21 ± 0.73	3.27 ± 0.76	0.779	0.819	0.006
hs-CRP (mg/L)	1.01 ± 0.96	0.69 ± 0.86	1.01 ± 1.02	1.07 ± 1.61	0.539	0.799	0.005

Data presented as mean ± SD

Abbreviations: hs-CRP, high-sensitivity C-reactive protein; Total chol, total cholesterol; Trig, triglyceride.

1 Between-group postintervention differences assessed using analysis of covariance adjusting for age, sex, BMI, baseline concentrations, and ω-3 supplement intervention Sig *q* < 0.05.

**TABLE 5:** The effect of ω-3 supplementation on blood lipid concentrations and hs-CRP.

	Supplement (*n =* 20)		Placebo (*n =* 20)		*P* value^1^	*q* value	Partial Eta squared
	Baseline	Postintervention	Baseline	Postintervention			
Total chol (mmol/L)	4.48 ± 0.97	4.33 ± 0.95	4.22 ± 0.63	4.42 ± 0.82	0.121	0.436	0.064
HDL (mmol/L)	1.46 ± 0.28	1.36 ± 0.26	1.34 ± 0.31	1.30 ± 0.31	0.514	0.734	0.017
LDL (mmol/L)	2.66 ± 1.05	2.19 ± 0.80	2.50 ± 0.66	2.43 ± 0.90	0.340	0.680	0.030
Trig (mmol/L)	0.90 ± 0.44	0.85 ± 0.53	0.73 ± 0.24	0.78 ± 0.27	0.161	0.459	0.054
TC:HDL	3.16 ± 0.85	3.24 ± 0.78	3.25 ± 0.66	3.51 ± 0.76	0.068	0.408	0.093
Trig:HDL	0.58 ± 0.28	0.67 ± 0.52	0.58 ± 0.28	0.65 ± 0.34	0.403	0.680	0.061
Non-HDL (mmol/L)	3.02 ± 0.95	2.96 ± 0.86	2.88 ± 0.58	3.11 ± 0.75	0.109	0.436	0.085
hs-CRP (mg/L)	1.14 ± 1.00	0.87 ± 1.08	0.88 ± 0.95	0.88 ± 1.50	0.907	0.907	0.000

Data presented as mean ± SD.

Abbreviations: hs-CRP, high-sensitivity C-reactive protein; Total chol, total cholesterol; Trig, triglyceride.

1 Between-group postintervention differences assessed using analysis of covariance adjusting for age, sex, BMI, baseline concentrations, and dietary fish intervention *q* < 0.05.

In the 4-arm sensitivity analysis, the combined fish + supplement group had significantly greater increases in erythrocyte EPA and significantly lower total n–6 PUFAs compared with the fish-only group (FDR *P* < 0.05) and greater increases in O3I, and DHA compared with the supplement-only group (FDR *P* < 0.05) (Supplemental Tables 3–5). No significant differences were observed between the combined group and either single-intervention group for any of the other outcomes. These results are consistent with the factorial analysis and did not alter the main interpretation of the intervention effects.

## Discussion

This study demonstrates that the consumption of 2 portions of fish per week or ω-3 supplements significantly increased the O3I and erythrocyte concentrations of n–3 PUFAs among low fish-consuming young adults with a low O3I at baseline. Furthermore, both interventions resulted in a significant decrease in erythrocyte total n–6 PUFAs. The consumption of 2 portions of fish per week also resulted in a significantly greater proportion of participants achieving an optimal O3I of >8%, which is considered to confer the greatest level of cardioprotection.

Current United Kingdom dietary recommendations, last updated in 2004, stipulate the consumption of 2 portions of fish per week (equivalent to 280 g/wk) with ≥1 portion being oily, which would contribute ∼450 mg EPA + DHA per day [4]. Although no formal recommendations for ω-3 supplementation currently exist, professional bodies have noted that a daily ω-3 supplement, equating to ∼500 mg EPA + DHA per day, is beneficial in increasing n–3 PUFA intakes among those who may exclude dietary sources, such as fish [9,10]. Our findings support existing United Kingdom dietary guidelines, demonstrating that both 2 portions of fish per week and a daily ω-3 supplement (providing 700 mg EPA + DHA) resulted in increasing the O3I by ∼2.0% over an 8-wk period among young healthy adults with low-habitual fish intake.

The findings of the current study are in alignment with, albeit limited previous research, which has shown both ω-3 supplementation and fish consumption to improve the O3I among various age groups. One previous smaller study of 23 premenopausal women aged 21–49 y also reported that the consumption of equal amounts of EPA and DHA from oily fish or fish oil capsules was equally effective in increasing RBC EPA and DHA [21]. Supplementation with EPA and DHA has been shown to improve the O3I in a dose-dependent manner, with reported increases typically ranging from 1.5% to 7.0% [13,22]. Studies using higher doses of ω-3 supplementation have shown greater increases, with 1000–1500 mg EPA + DHA improving the O3I by ≤5% regardless of baseline status [[22], [23], [24]]. Benefits have also been observed with more conservative doses over a relatively short supplementation period [25]. In this study, although a higher proportion of participants in the supplement group achieved an O3I >8% (indicative of low CVD risk) relative to the placebo group, this difference did not reach statistical significance. Notably, a longer study among healthy adults aged 20–40 y reported that 600 mg/d of EPA + DHA over 5 mo increased O3I from 4.38% to 6.82% [13]. Although variations in study compliance within these studies may have led to lower-than-expected increases in the O3I [26], these findings may also suggest that although moderate doses of ω-3 supplementation may improve the O3I, they may not be sufficient to consistently achieve optimal levels, implying that either a higher dose or a longer duration of supplementation may be necessary to achieve an O3I above 8%. Fewer intervention studies have investigated the effect of fish consumption on the O3I. One small study showed an increase of 1.01% in the O3I among adults with coronary artery disease after the consumption of 150 g oily fish twice per week for 12 wk [8]. Another larger Norwegian study of 478 adolescents reported a smaller increase of 0.6% among those consuming 80–100 g of fish 3 times per week for 12 wk [15]. The variations in the O3I observed between fish-based dietary interventions may be owing to differences in study compliance, variations in the amount of PUFA supplied by the fish intervention, genetic differences influencing PUFA metabolism, and variations in habitual fish intakes between populations [27]. In addition, in the current study, participants had a mean baseline O3I of ∼5.0%, which is lower than the previously mentioned studies, potentially suggesting that increases may be more pronounced among those with lower baseline status owing to being low consumers of fish.

Numerous studies have highlighted the benefits of a higher O3I, particularly in relation to CVD risk reduction, with epidemiologic evidence indicating a 34% reduction in all-cause mortality and a 39% reduction in incident CVD among individuals with higher a O3I [28]. Nevertheless, population-level data on the O3I in the United Kingdom and Ireland remain limited. Recent studies have, however, suggested that the majority of the population have an O3I of ∼5.58%, which is below the optimal of 8.0% [12,29]. This estimated low population status has largely been attributed to low levels of fish consumption and no set recommendations for ω-3 supplementation [30]. Young adults tend to have one of the lowest rates of fish consumption within the population, placing them at particular risk of low n–3 PUFA intakes during a period when vascular changes associated with atherosclerosis may begin to occur [6,31]. In the present study, participants had a mean baseline O3I of 5.0%, which is generally reflective of a population who are nonfish consumers [32]. Following dietary intervention with fish, the proportion of participants in the low-risk O3I category (>8%) was significantly greater when compared with those in the no fish group. These findings highlight the efficacy of the current recommendations for fish consumption in improving the O3I and provide promising evidence of early dietary interventions, which shift individuals into an O3I category associated with a lower risk for CVD. In addition, the similar increase within the supplement group highlights alternative strategies for improving the O3I and lowering CVD risk for those who may exclude fish within their diet; however, consideration should be given to the additional nutritional benefits of fish as a whole food, which may not be achieved through supplementation alone [33].

Although both interventions resulted in a similar increase in the O3I and total erythrocyte n–3 PUFA, the differing effects on the individual n–3 PUFAs are noteworthy. In this study, the fish intervention significantly increased DHA but had no significant effect on EPA, whereas supplementation significantly increased EPA with no change in DHA. Although the combined intake of EPA and DHA is known to support overall health, recent attention has shifted toward their distinct physiological roles [34]. Some studies suggest that EPA may be more effective in reducing the risk of major cardiovascular events [35], whereas DHA may exert a stronger triglyceride-lowering effect [34]. Although dependent on the species of fish, generally, fish tend to be higher in DHA than EPA, whereas the amount of EPA/DHA in commercial supplements varies but typically is higher in EPA [36]. Given desaturation of EPA to DHA is relatively low in vivo, the increases observed within groups may suggest a more pronounced effect of fish on DHA concentrations, whereas supplementation may increase EPA concentrations to a greater extent. Future research may wish to directly compare the effects of fish and ω-3 supplementation of EPA + DHA concentration owing to their proposed differential health effects.

In addition to increasing the O3I and erythrocyte n–3 PUFA concentrations, both fish consumption and ω-3 supplementation resulted in lower total n–6 PUFA concentrations, potentially suggesting a shift toward a more favorable fatty acid profile. Two studies investigating the effects of a much higher dose of 3–4 g/day EPA + DHA also reported a significant reduction in n–6 PUFAs [37,38]. Similarly, evidence from a large number of studies supports a lowering effect of fish on n–6 PUFAs [[39], [40], [41]]. Both n–3 and n–6 PUFAs share enzymatic pathways resulting in a competition for enzymes, with n–3 PUFAs often being incorporated into the phospholipid membrane at the expense of the n–6 PUFAs, which likely explains the apparent decrease often reported following consumption of fish and/or ω-3 supplementation [41]. The n–6 PUFAs are generally, although not always, associated with a more proinflammatory immune response [42]. Recent evidence has however reported inverse associations between inflammatory markers and n–6 PUFAs [43]. Future research is needed to determine the clinical significance of reductions in n–6 PUFAs following fish or ω-3 supplementation.

Despite both fish consumption and ω-3 supplementation increasing the O3I, there were no significant differences in lipid profiles or hs-CRP between groups. Previous research has emphasized the anti-inflammatory and lipid-modifying properties of n–3 PUFAs [44]. One larger study investigating the effect of 1800 mg EPA + DHA on lipid profiles among those with high CVD risk noted significant increases in HDL [45], whereas other studies have shown reductions in triglycerides and inflammatory markers including CRP using supplementation >1 g EPA + DHA per day among normolipidemic and hyperlipidemic individuals [46,47]. Studies investigating the effects of fish have shown similar findings, with evidence now also supporting a role for fish consumption in lowering triglyceride concentrations [41,48]. The participants of the current study were healthy individuals and studies that have reported an effect of either fish or ω-3 supplementation among normolipidemic individuals typically use doses, which are much higher than what would be supplied within the current study [49]. In addition, although 8 wk is sufficient to observe a change in the O3I, a longer duration may be required to elicit meaningful changes in lipid profiles or hs-CRP when using modest doses of n–3 PUFA and may warrant further investigation.

This study investigated the effects of fish consumption and ω-3 supplementation, aligned to current dietary recommendations, on the O3I. Participants were low-fish consumers and thus had a low baseline O3I, allowing for evaluation of how population dietary guidelines may influence the O3I and, subsequently, CVD risk. The target population within this study was also young adults, a population often underrepresented in relation to CVD research. This study does, however, have several limitations; the majority of participants within the current study were female (*n*= 29, 72.5%) and therefore may not be representative of the entire population and results should be confirmed among other population groups. As mentioned, the present study was a relatively short-term intervention, and thus results cannot be extrapolated to long-term consumption. A longer-term intervention may provide a greater insight into the maximum changes in the O3I resulting from fish consumption or supplementation. Although increasing the O3I into a lower risk category can have a clinically significant impact with regard to CVD risk, the current study only assessed potential changes in lipid profiles and hs-CRP as additional measures of CVD. Future research may wish to include other markers of cardiovascular and endothelial function such as flow-mediated dilation and blood pressure to gain a full depiction of the impact of the dietary guidance for fish consumption and supplementation on CVD risk among young adults.

In conclusion, this study demonstrates that the consumption of the recommended 2 portions of fish per week or ω-3 supplementation can result in a significant increase in the O3I, which may contribute to a reduced risk of CVD among low fish-consuming young adults. Furthermore, both interventions resulted in shifts toward more favorable erythrocyte fatty acid profiles by lowering total n–6 PUFAs. These findings support the efficacy of current public health recommendations for fish consumption and ω-3 intake and highlight the potential for early dietary interventions to reduce CVD risk among young adults. Future research is needed to evaluate the long-term effectiveness of these interventions in achieving and maintaining an optimal O3I of >8%. Neither fish consumption nor ω-3 supplementation was found to have significant effects on blood lipid profiles or hs-CRP, which may be owing to the majority of participants being within the recommended clinical ranges, the modest dose of n–3 PUFAs supplied in this study and the relatively short intervention duration. Future longer-term interventions, which incorporate additional markers of cardiovascular and endothelial function are warranted to explore the longer-term impact of current dietary guidance for fish and ω-3 consumption on the O3I and subsequent CVD risk.

## Author contributions

The authors’ responsibilities were as follows – JEM, AJY, MSM, PJA, JJS, EMM: designed research; JEM, RAK: conducted research; JEM: analyzed data and performed statistical analysis; JEM: wrote paper; EMM, AJY, MSM, PJA, JJS, EvW: reviewed manuscript; EMM had primary responsibility for final content; and all authors: read and approved the final manuscript.

## Data availability

Data described in the manuscripts, code book, and analytic code will be made available on request pending.

## Funding

JEM is a PhD Student funded by Department for the Economy scholarship, Ulster University. EPAX Norway provided the supplements and Lir Native Seafood and Scran, Coleraine provided the fish used within the intervention. Only the named authors had input to the study design, data interpretation, and study conclusions.

## Conflict of interest

EMM reports equipment, drugs, or supplies was provided by Epax Norway AS. EMM reports equipment, drugs, or supplies was provided by Lir Native Seafood and Scran. All other authors report no conflicts of interest.

## References

[1] Harris W.S., Tintle N.L., Etherton M.R., Vasan R.S. (2018). Erythrocyte long-chain omega-3 fatty acid levels are inversely associated with mortality and with incident cardiovascular disease: the Framingham heart study. J. Clin. Lipidol..

[2] Harris W.S., Von Schacky C. (2004). The omega-3 index: a new risk factor for death from coronary heart disease. Prev. Med..

[3] European Food Safety Authority (2010). Scientific opinion on dietary reference values for fats, including saturated fatty acids, polyunsaturated fatty acids, monounsaturated fatty acids, trans fatty acids, and cholesterol. EFSA J.

[4] Scientific Advisory Committee on Nutrition (2004).

[5] Public Health England, NDNS: results from years 9 to 11 (combined)-report [Internet], Last updated 2020 [Cited May 2025] https://www.gov.uk/government/statistics/ndns-results-from-years-9-to-11-2016-to-2017-and-2018-to-2019.

[6] Derbyshire E. (2019). Oily fish and omega-3s across the life stages: a focus on intakes and future directions. Front. Nutr..

[7] Govzman S., Looby S., Wang X., Butler F., Gibney E.R., Timon C.M. (2021). A systematic review of the determinants of seafood consumption. Br. J. Nutr..

[8] Brazionis L., Ting E., Itsiopoulos C., Wilson A., Hodge A. (2012). The effects of fish or fish oil on the omega-3 index. Nutr. Diet..

[9] Food Standards Agency of Ireland, Scientific recommendations for food-based dietary guidelines for older adults in Ireland [Internet], Last updated 2021 [Cited May 2025] https://www.fsai.ie/getmedia/c0610e7f-9bfa-457a-9dca-3f97149e43a1/scientific-recommendations-for-food-based-dietary-guidelines-for-older-adults-in-ireland.pdf?ext=.pdf.

[10] British Dietetic Association (2021). https://www.bda.uk.com/resource/omega-3.html.

[11] Salisbury A.C., Amin A.P., Harris W.S., Chan P.S., Gosch K.L., Rich M.W. (2011). Predictors of omega-3 index in patients with myocardial infarction. Mayo Clin. Proc..

[12] Schuchardt J.P., Tintle N., Westra J., Harris W.S. (2023). Estimation and predictors of the Omega-3 Index in the UK Biobank. Br. J. Nutr..

[13] Flock M.R., Skulas-Ray A.C., Harris W.S., Etherton T.D., Fleming J.A., Kris-Etherton P.M. (2013). Determinants of erythrocyte omega-3 fatty acid content in response to fish oil supplementation: a dose-response randomized controlled trial. J. Am. Heart. Assoc..

[14] Macartney M.J., Hingley L., Brown M.A., Peoples G.E., McLennan P.L. (2014). Intrinsic heart rate recovery after dynamic exercise is improved with an increased omega-3 index in healthy males. Br. J. Nutr..

[15] Handeland K., Skotheim S., Baste V., Graff I.E., Froyland L., Lie O. (2018). The effects of fatty fish intake on adolescents’ nutritional status and associations with attention performance: results from the FINS-TEENS randomized controlled trial. Nutr. J..

[16] Public Health England (2025). https://www.gov.uk/government/publications/composition-of-foods-integrated-dataset-cofid.

[17] Harris W.S., Polreis J. (2016). Measurement of the omega-3 index in dried blood spots. Ann. Clin. Lab. Res.

[18] Yeatts S.D., Martin R.H. (2015). What is missing from my missing data plan?. Stroke.

[19] Jakobsen J.C., Gluud C., Wetterslev J., Winkel P. (2017). When and how should multiple imputation be used for handling missing data in randomised clinical trials—a practical guide with flowcharts. BMC Med. Res. Methodol..

[20] Conway M.C., McSorley E.M., Mulhern M.S., Spence T., Wijngaarden E.V., Watson G.E. (2021). The influence of fish consumption on serum n-3 polyunsaturated fatty acid (PUFA) concentrations in women of childbearing age: a randomised controlled trial (the iFish Study). Eur. J. Nutr..

[21] Harris W.S., Pottala J.V., Sands S.A., Jones P.G. (2007). Comparison of the effects of fish and fish-oil capsules on the n-3 fatty acid content of blood cells and plasma phospholipids. Am. J. Clin. Nutr..

[22] Dempsey M., Rockwell M.S., Wentz L.M. (2023). The influence of dietary and supplemental omega-3 fatty acids on the omega-3 index: a scoping review. Front. Nutr..

[23] Hooper C., De Souto Barreto P., Coley N., Cantet C., Cesari M., Andrieu S. (2017). Cognitive changes with omega-3 polyunsaturated fatty acids in nondemented older adults with low omega-3 index. J. Nutr. Health Aging.

[24] West A.L., Kindberg G.M., Hustvedt S.O., Calder P.C. (2018). A novel self-micro-emulsifying delivery system enhances enrichment of eicosapentaenoic acid and docosahexaenoic acid after single and repeated dosing in healthy adults in a randomized trial. J. Nutr..

[25] Hingley L., Macartney M.J., Brown M.A., McLennan P.L., Peoples G.E. (2017). DHA-rich fish oil increases the omega-3 index and lowers the oxygen cost of physiologically stressful cycling in trained individuals. Int. J. Sport Nutr. Exerc. Metab..

[26] Marriott B.P., Turner T.H., Hibbeln J.R., Newman J.C., Pregulman M., Malek A.M. (2021). Impact of fatty acid supplementation on cognitive performance among United States (US) military officers: the ranger resilience and improved performance on phospholipid-bound omega-3’s (RRIPP-3) study. Nutrients.

[27] Wu W.C., Wu P.Y., Chan C.Y., Lee M.F., Huang C.Y. (2023). Effect of FADS1 rs174556 genotype on polyunsaturated fatty acid status: a systematic review and meta-analysis. Adv. Nutr..

[28] Harris W.S., Del Gobbo L., Tintle N.L. (2017). The omega-3 index and relative risk for coronary heart disease mortality: estimation from 10 cohort studies. Atherosclerosis.

[29] Stark K.D., Elswyk M.E.V., Higgins M.R., Weatherford C.A., Salem N. (2016). Global survey of the omega-3 fatty acids, docosahexaenoic acid and eicosapentaenoic acid in the blood stream of healthy adults. Prog. Lipid. Res.

[30] Jackson K.H., Polreis J.M., Tintle N.L., Kris-Ehterton P.M., Harris W.S. (2019). Association of reported fish intake and supplementation status with the omega-3 index. Prostaglandins Leukot. Essent. Fatty Acids.

[31] Poznyak A.V., Yakovlev A.A., Popov M.А., Zhigmitova E.B., Sukhorukov V.N., Orekhov A.N. (2024). Atherosclerosis originating from childhood: specific features. J. Biomed. Res..

[32] Schuchardt J.P., Beinhorn P., Hu X.F., Chan H.M., Roke K., Bernasconi A. (2024). Omega-3 world map:2024 update. Prog. lipid res..

[33] Chen J., Jayachandran M., Bai W., Xu B. (2022). A critical review on the health benefits of fish consumption and its bioactive constituents. Food Chem..

[34] Choi G.Y., Calder P.C. (2024). The differential effects of eicosapentaenoic acid and docosahexaenoic acid on cardiovascular risk factors: an updated systematic review of randomized controlled trials. Front. Nutr..

[35] Toth P.P., Chapman M.J., Parhofer K.G., Nelson J.R. (2022). Differentiating EPA from EPA/DHA in cardiovascular risk reduction. Am. Heart J. Plus..

[36] Yi T., Li S.M., Fan J.Y., Fan L.L., Zhang Z.F., Luo P (2014). Comparative analysis of EPA and DHA in fish oil nutritional capsules by GC-MS. Lipids Health Dis.

[37] Mieszkowski J., Konert M., Kochanowicz A., Niespodziński B., Brzezińska P., Stankiewicz B. (2024). Supplementation with n-3 polyunsaturated fatty acids does not impact physical performance but affects n-6 polyunsaturated fatty acid levels. J. Funct. Foods..

[38] Grytten E., Laupsa-Borge J., Cetin K., Bohov P., Nordrehaug J.E., Skorve J. (2025). Inflammatory markers after supplementation with marine n-3 or plant n-6 PUFAs: a randomized double-blind crossover study. J. Lipid. Res..

[39] Raatz S.K., Rosenberger T.A., Johnson L.K., Wolters W.W., Burr G.S., Picklo M.J. (2013). Dose-dependent consumption of farmed Atlantic salmon (Salmo salar) increases plasma phospholipid n-3 fatty acids differentially. J. Acad. Nutr. Diet..

[40] Grieger J.A., Miller M.D., Cobiac L. (2014). Investigation of the effects of a high fish diet on inflammatory cytokines, blood pressure, and lipids in healthy older Australians. Food. Nutr. Res..

[41] McMullan J.E., Yeates A.J., Allsopp P.J., Mulhern M.S., Strain J.J., van Wijngaarden E. (2023). Fish consumption and its lipid modifying effects – a review of intervention studies. Neurotoxicology.

[42] Calder P.C. (2010). Omega-3 fatty acids and inflammatory processes. Nutrients.

[43] Lai H.T.M., Ryder N.A., Tintle N.L., Jackson K.H., Kris-Etherton P.M., Harris W.S. (2025). Red blood cell omega-6 fatty acids and biomarkers of inflammation in the Framingham Offspring Study. Nutrients.

[44] Calder P.C. (2020). n-3 PUFA and inflammation: from membrane to nucleus and from bench to bedside. Proc. Nutr. Soc..

[45] Cartolano F.D.C., Dias G.D., Miyamoto S., Damasceno N.R.T. (2021). Omega-3 fatty acids improve functionality of high-density lipoprotein in individuals with high cardiovascular risk: a randomised parallel controlled and double-blind clinical trial. Front. Nutr..

[46] Leslie M.A., Cohen D.J., Liddle D.M., Robinson L.E., Ma D.W. (2015). A review of the effect of omega-3 polyunsaturated fatty acids on blood triacylglycerol levels in normolipidemic and borderline hyperlipidemic individuals. Lipids Health Dis.

[47] Kavyani Z., Musazadeh V., Fathi S., Hossein Faghfouri A., Dehghan P., Sarmadi B. (2022). Efficacy of the omega-3 fatty acids supplementation on inflammatory biomarkers: an umbrella meta-analysis. Int. Immunopharmacol..

[48] Zibaeenezhad M.J., Ghavipisheh M., Attar A., Aslani A. (2017). Comparison of the effect of omega-3 supplements and fresh fish on lipid profile: a randomized, open-labelled trial, Nutr. Diabetes.

[49] Wang T., Zhang X., Zhou N., Shen Y., Li B., Chen B.E. (2023). Association between omega-3 fatty acid intake and dyslipidemia: a continuous dose-response meta-analysis of randomized controlled trials. J. Am. Heart Assoc..

