# Supplementary material for: Influence of Fish Consumption and ω-3 Supplementation on the ω-3 Index of Young Adults: A 2 × 2 Factorial Randomized Controlled Trial (YouFish Study)
Source: J Nutr. 2025 Oct 10;155(12):4345–55. doi: 10.1016/j.tjnut.2025.10.010 (PMC12799427; doi:10.1016/j.tjnut.2025.10.010)
Supplement: Multimedia component 1 [file mmc1.docx]

| **Supplementary Table 1: Estimated amount of n-3 PUFA (g/week) provided by fish intervention and omega-3 supplement** | | | |
| --- | --- | --- | --- |
|  | EPA (g/week) | DHA (g/week) | EPA + DHA (g/week) |
| Fish intervention | 0.83 | 1.57 | 2.40 |
| Omega-3 supplements | 2.80 | 2.10 | 4.90 |
| n-3 PUFA intakes from fish were estimated based on published nutrient composition data. | | | |
