# Supplementary material for: Influence of Fish Consumption and ω-3 Supplementation on the ω-3 Index of Young Adults: A 2 × 2 Factorial Randomized Controlled Trial (YouFish Study)
Source: J Nutr. 2025 Oct 10;155(12):4345–55. doi: 10.1016/j.tjnut.2025.10.010 (PMC12799427; doi:10.1016/j.tjnut.2025.10.010)
Supplement: Multimedia component 2 [file mmc2.docx]

| **Supplementary Table 2:** Dietary intake of macronutrients and PUFA at baseline according to intervention group | | | | | | | |
| --- | --- | --- | --- | --- | --- | --- | --- |
|  | **Fish (n=20)** | **No Fish (n=20)** | **p-value*** | **Supplement (n=20)** | **Placebo (n=20)** | **p-value*=** | **q-value*** |
| Energy (kcal/day) | 1817 (1270, 2119) | 1796 (1368, 1992) | 0.753 | 1484 (1193, 1866) | 1912 (1766, 2202) | **0.008** | 0.051 |
| Energy (kJ/day) | 7638 (5334, 8896) | 7554 (5737, 8342) | 0.730 | 6225 (5014, 7834) | 8039 (7413, 9251) | **0.008** | 0.051 |
| Protein (g/day) | 82.8 (66.1, 105.5) | 80.5 (63.6, 116.6) | 0.988 | 71.6 (62.2, 83.0) | 98.5 (71.9, 129.6) | **0.007** | 0.051 |
| Protein (%energy) | 19.3 (18.2, 22.6) | 22.9 (17.1, 24.4) | 0.221 | 19.3 (17.6, 23.0) | 22.2 (17.9, 24.0) | 0.443 | 0.495 |
| Fat (g/day) | 64.5 (51.9, 82.7) | 71.7 (56.4, 79.8) | 0.845 | 57.9 (47.1, 78.5) | 75.3 (17.9, 85.2) | **0.017** | 0.065 |
| Fat (%energy) | 34.5 (32.7, 36.8) | 37.4 (32.9, 39.4) | 0.150 | 36.7 (32.7, 37.9) | 35.6 (33.1, 39.1) | 0.988 | 0.988 |
| Carbohydrates (g/day) | 210.6 (136.1, 248.2) | 195.6 (126.2, 224.7) | 0.313 | 152.1 (115.5, 211.5) | 212.6 (178.9, 235.4) | 0.066 | 0.171 |
| Carbohydrates (%energy) | 45.4 (41.2, 48.1) | 41.0 (37.3, 46.7) | 0.086 | 43.1 (39.8, 50.1) | 43.1 (38.0, 47.5) | 0.558 | 0.589 |
| Saturated fat (g/day) | 24.1 (18.9, 31.1) | 23.3 (18.2, 27.2) | 0.799 | 20.8 (18.1, 26.4) | 26.2 (20.1, 33.4) | 0.098 | 0.186 |
| Linoleic Acid (g/day) | 0.16 (0.04, 0.34) | 0.12 (0.05, 0.69) | 0.935 | 0.12 (0.04, 0.31) | 0.26 (0.05, 0.79) | 0.386 | 0.479 |
| Arachidonic Acid (g/day) | 0.06 (0.03, 0.09) | 0.03 (0.01, 0.06) | 0.051 | 0.04 (0.01, 0.08) | 0.04 (0.02, 0.08) | 0.403 | 0.479 |
| Omega-6 (Total) | 3.33 (1.96, 5.62) | 3.67 (2.05, 7.05) | 0.620 | 3.11 (1.96, 4.57) | 5.03 (2.35, 6.80) | 0.233 | 0.341 |
| Alpha Linoleic Acid (g/day) | 0.21 (0.14, 0.49) | 0.18 (0.10, 0.36) | 0.415 | 0.18 (0.10, 0.27) | 0.27 (0.11, 0.68) | 0.208 | 0.329 |
| Eicosapentaenoic Acid (g/day) | 0.01 (0.01, 0.02) | 0.01 (0.00, 0.01) | 0.059 | 0.01 (0.00, 0.01) | 0.02 (0.01, 0.03) | **0.014** | 0.065 |
| Docosahexaenoic Acid (g/day) | 0.03 (0.02, 0.05) | 0.02 (0.01, 0.03) | 0.135 | 0.02 (0.01, 0.03) | 0.03 (0.02, 0.05) | 0.144 | 0.249 |
| Omega-3 (Total) | 0.72 (0.30, 1.92) | 0.49 (0.34, 0.91) | 0.799 | 0.46 (0.33, 0.93) | 0.72 (0.26, 2.16) | 0.391 | 0.479 |
| MUFA (g/day) | 14.06 (10.66, 23.09) | 18.47 (11.70, 21.46) | 0.480 | 13.77 (10.93, 18.47) | 20.11 (13.63, 26.02) | **0.029** | 0.092 |
| PUFA (g/day) | 9.03 (3.83, 12.63) | 8.42 (5.33, 10.60) | 0.799 | 7.33 (3.99, 9.85) | 10.07 (6.23, 12.40) | 0.081 | 0.171 |
| Cholesterol | 224.21 (135.39, 378.41) | 277.23 (176.30, 356.63) | 0.499 | 210.27 (147.93, 298.15) | 312.23 (172.24, 555.20) | 0.075 | 0.171 |
| Data expressed as median (IQR), where IQR is the 25^th^ and 75^th^ centile. MUFA; monounsaturated fatty acids, PUFA; polyunsaturated fatty acids.  *p-value for significant difference between intervention groups at baseline as determined using Mann Whitney U test; False discovery rate (FDR) correction (Benjamini–Hochberg) was applied within each comparison; for the fish vs. no fish comparison, all adjusted p-values exceeded 0.5. q<0.005 considered significant after FDR correction for multiple testing | | | | | | | |

| **Supplementary Table 3: Characteristics of YouFish study participants at baseline according to intervention group** | | | | |
| --- | --- | --- | --- | --- |
|  | **No Fish and Placebo (n=10)** | **Omega 3 supplement and no fish (n=10)** | **Fish and omega 3 supplement (n=10)** | **Fish and Placebo (n=10)** |
| Age (years) | 20.5 (19.0, 24.50) | 22.5 (21.0, 25.3) | 21.0 (19.0, 25.5) | 21.0 (20.8, 23.5) |
| Weight (kg) | 71.5 (64.0, 84.6) | 62.40 (57.7, 85.1) | 65.7 (58.90, 73.35) | 64.7 (58.0, 75.3) |
| Height (m) | 1.70±0.08 | 1.68±0.04 | 1.67±0.10 | 1.71±0.09 |
| BMI (kg/m2) | 25.13±3.69 | 24.52±4.73 | 24.12±2.83 | 22.79±2.56 |
| Body Fat (%) | 16.8±9.58 | 14.54±6.32 | 16.61±7.09 | 13.95±7.47 |
| *Gender* |  |  |  |  |
| Female | 6 (60.0) | 7 (70.0) | 9 (90.0) | 7 (70.0) |
| Male | 4 (40.0) | 3 (30.0) | 1 (10.0) | 3 (30.0) |
| *Consumes Alcohol* |  |  |  |  |
| Yes | 8 (80.0) | 9 (90.0) | 9 (90.0) | 4 (40.0) |
| No | 2 (20.0) | 1 (10.0) | 1 (10.0) | 6 (60.0) |
| *Smoker* |  |  |  |  |
| Yes | 1 (10.0) | 0 (0.0) | 0 (0.0) | 0 ((0.0) |
| No | 9 (90.0) | 10 (100.0) | 10 (100.0) | 10 (100.0) |
| O3I (%) | 5.11±0.63 | 5.13±0.51 | 4.99±0.64 | 5.01±0.71 |
| ALA (%) | 0.16 (0.13, 0.22) | 0.16 (0.13, 0.21) | 0.18 (0.15, 0.23) | 0.22 (0.13, 0.51) |
| EPA (%) | 0.52 (0.47, 0.66) | 0.63 (0.52, 0.81) | 0.53 (0.48, 0.67) | 0.60 (0.42, 0.71) |
| DPA (%) | 2.89 (2.40, 2.98) | 2.93 (2.56, 3.36) | 2.79 (2.45, 3.07) | 2.70 (1.31, 3.00) |
| DHA (%) | 4.43 (3.46, 4.80) | 4.43 (3.99, 4.99) | 4.18 (3.77, 4.79) | 3.53 (2.88, 4.84) |
| Total n-3 (%) | 7.90 (6.93, 8.50) | 8.23 (7.90, 8.69) | 7.87 (7.14, 8.44) | 6.80 (2.95, 8.55) |
| *O3I Category* |  |  |  |  |
| High (<4%) | 0 (0.0) | 0 (0.0) | 0 (0.0) | 2 (20.0) |
| Medium (4-8%) | 10 (100.0) | 10 (100.0) | 10 (100.0) | 8 (80.0) |
| Low (>8%) | 0 (0.0) | 0 (0.0) | 0 (0.0) | 0 (0.0) |
| Data presented as Mean±SD or Median (IQR), where IQR is the 25th and 75th percentile; or n (%) where appropriate. BMI, Body mass index; O3I, Omega-3 index, EPA, Eicosapentaenoic acid, DPA, Docosapentaenoic acid, DHA, Docosahexaenoic acid *p-value for significant difference between intervention groups at baseline as determined using One-Way Anova test for parametric data and Kruskal Wallis test for non parametric data, or Fishers Exact as appropriate; p<0.05 considered significant (all p>0.05, with the exception of alcohol consumption (p = 0.036) | | | | |

| **Supplementary Table 4: The effect of omega-3 supplementation and fish on Omega-3 index and red blood cell fatty acid concentrations** | | | | | | | | | | | | | | | | | | |  |
| --- | --- | --- | --- | --- | --- | --- | --- | --- | --- | --- | --- | --- | --- | --- | --- | --- | --- | --- | --- |
|  | **Placebo and no fish (n=10)** | | |  | | **Fish and placebo (n=10)** | |  | **Omega 3 supplement and no fish (n=10)** | |  | **Fish and omega 3 supplement (n=10)** | |  |  |  | |  |  |
|  | **Baseline** | **Post-intervention** |  | | **Baseline** | | **Post-intervention** |  | **Baseline** | **Post-intervention** |  | **Baseline** | **Post-intervention** | **P-value** | **q-value** | | **Partial Eta Squared** | | |
| O3I (%) | 5.11±0.63 | 5.12±0.81^a^ |  | | 5.01±0.71 | | 6.98±1.36^bc^ |  | 5.13±0.51 | 6.62±0.68^b^ |  | 4.49±0.64 | 7.57±0.80^c^ | **<0.001** | **<0.001** | | 0.600 | | |
| LA (%) | 13.65±3.34 | 13.08±3.17^a^ |  | | 16.24±4.78 | | 12.57±1.74^b^ |  | 12.21±1.35 | 11.63±1.24^b^ |  | 13.24±3.86 | 11.45±1.11^b^ | **<0.001** | **<0.001** | | 0.440 | | |
| AA (%) | 16.35±2.77 | 16.86±2.48 |  | | 14.48±2.70 | | 16.42±1.26 |  | 17.27±0.85 | 16.83±1.20 |  | 16.94±1.95 | 16.70±0.72 | 0.714 | 0.785 | | 0.041 | | |
| ALA (%) | 0.19±0.08 | 0.18±0.05 |  | | 0.38±0.41 | | 0.17±0.05 |  | 0.17±0.05 | 0.17±0.04 |  | 0.23±0.17 | 0.15±0.03 | 0.256 | 0.313 | | 0.117 | | |
| EPA (%) | 0.55±0.10 | 0.63±0.18^a^ |  | | 0.57±0.16 | | 0.95±0.33^ab^ |  | 0.63±0.16 | 1.32±0.36^c^ |  | 0.56±0.13 | 1.37±0.38^c^ | **<0.001** | **<0.001** | | 0.509 | | |
| DPA (%) | 2.61±0.76 | 2.77±0.70 |  | | 2.32±0.84 | | 3.02±0.23 |  | 2.94±0.42 | 3.48±0.31 |  | 2.66±0.58 | 3.14±0.29 | **0.042** | 0.058 | | 0.223 | | |
| DHA (%) | 4.20±0.87 | 4.40±0.92^a^ |  | | 3.80±0.98 | | 6.01±1.08^bc^ |  | 4.48±0.53 | 5.30±0.46^b^ |  | 4.26±0.70 | 6.20±0.56^c^ | **<0.001** | **<0.001** | | 0.476 | | |
| Total n-6 | 36.10±1.44 | 36.39±1.51^a^ |  | | 36.43±2.49 | | 35.13±1.60^b^ |  | 36.35±1.09 | 34.92±1.37^bc^ |  | 37.06±1.42 | 34.48±1.35^c^ | **<0.001** | **<0.001** | | 0.420 | | |
| Total n-3 | 7.54±1.47 | 7.98±1.53^a^ |  | | 7.07±1.43 | | 10.16±1.45^b^ |  | 8.21±0.55 | 10.26±0.72^b^ |  | 7.71±0.94 | 10.85±0.93^b^ | **<0.001** | **<0.001** | | 0.467 | | |
| AA:LA ratio | 1.28±0.39 | 1.36±0.35 |  | | 0.99±0.41 | | 1.33±0.24 |  | 1.43±0.22 | 1.47±0.24 |  | 1.37±0.35 | 1.47±0.17 | 0.867 | 0.857 | | 0.022 | | |
| EPA:ALA ratio | 3.37±1.23 | 3.56±0.92^a^ |  | | 2.79±2.05 | | 6.02±2.72^b^ |  | 4.18±1.8 | 8.14±2.78^bc^ |  | 3.04±1.31 | 9.80±3.34^c^ | **<0.001** | **<0.001** | | 0.540 | | |
| Data presented as Mean±SD  O3I: Omega-3 index, LA: Linoleic acid, AA: Arachidonic acid, ALA: Alpha linoleic Acid, EPA: Eicosapentaenoic acid, DPA: Docosapentaenoic acid, DHA: Docosahexaenoic acid *Between-group post-intervention differences assessed using Analysis of Covariance adjusting for age, sex, BMI, baseline concentrations, Sig q<0.05 Different superscripts denotes statistical significance between groups | | | | | | | | | | | | | | | | | | |  |

| **Supplementary Table 5: The effect of omega-3 supplementation and fish on blood lipid concentrations and hs-CRP** | | | | | | | | | | | | | | | |
| --- | --- | --- | --- | --- | --- | --- | --- | --- | --- | --- | --- | --- | --- | --- | --- |
|  | **Placebo and no fish (n=10)** | |  | **Fish and placebo (n=10)** | |  | **Omega 3 supplement and no fish (n=10)** | |  | **Fish and omega 3 supplement (n=10)** | |  |  |  |  |
|  | **Baseline** | **Post-intervention** |  | **Baseline** | **Post-intervention** |  | **Baseline** | **Post-intervention** |  | **Baseline** | **Post-intervention** | **P-value** | **q-value** | **Partial Eta Squared** |  |
| Total Chol (mmol/l) | 4.43±0.82 | 4.79±0.94 |  | 4.01±0.29 | 4.04±0.45 |  | 4.63±0.71 | 4.27±0.60 |  | 4.33±1.21 | 4.38±1.24 | 0.072 | 0.192 | 0.194 |  |
| HDL(mmol/l) | 1.27±0.24 | 1.24±0.27 |  | 1.41±0.36 | 1.37±0.36 |  | 1.36±0.25 | 1.28±0.27 |  | 1.56±0.26 | 1.44±0.25 | 0.906 | 0.965 | 0.017 |  |
| LDL (mmol/l) | 2.79±0.75 | 2.85±0.85 |  | 2.22±0.41 | 2.00±0.47 |  | 2.97±0.85 | 2.27±0.63 |  | 2.35±1.18 | 2.11±0.97 | 0.066 | 0.192 | 0.198 |  |
| Trig (mmol/l) | 0.79±0.25 | 0.95±0.26 |  | 0.66±0.22 | 0.60±0.11 |  | 1.06±0.52 | 0.97±0.61 |  | 0.74±0.28 | 0.72±0.44 | 0.493 | 0.357 | 0.071 |  |
| TC:HDL | 3.52±0.53 | 3.93±0.59 |  | 2.98±0.68 | 3.10±0.70 |  | 3.52±0.90 | 3.47±0.94 |  | 2.79±0.64 | 3.01±0.53 | 0.369 | 0.590 | 0.092 |  |
| Trig:HDL | 0.66±0.33 | 0.83±0.39 |  | 0.50±0.20 | 0.46±0.14 |  | 0.85±0.52 | 0.85±0.67 |  | 0.48±0.16 | 0.49±0.22 | 0.258 | 0.516 | 0.117 |  |
| non-HDL (mmol/l) | 3.15±0.67 | 3.56±0.78 |  | 2.60±0.30 | 2.67±0.38 |  | 3.27±0.78 | 2.99±0.66 |  | 2.78±1.08 | 2.93±1.06 | 0.056 | 0.192 | 0.208 |  |
| hs-CRP (mg/l) | 0.95±1.13 | 1.27±2.08 |  | 0.82±0.79 | 0.49±0.31 |  | 1.08±0.96 | 0.86±1.03 |  | 1.20±1.10 | 0.88±1.18 | 0.965 | 0.965 | 0.008 |  |
| Data presented as Mean±SD  Trig: Triglyceride, Total chol: total cholesterol, HDL: High density lipoprotein, LDL: Low density Lipoprotein; hs-CRP: high-sensitivity C-reactive protein  *Between-group post-intervention differences assessed using Analysis of Covariance adjusting for age, sex, BMI, baseline concentrations, Sig q<0.05 Different superscripts denotes statistical significance between groups. | | | | | | | | | | | | | | | |
